# Technological limitations in obtaining and using cellulose biocomposites

**DOI:** 10.3389/fbioe.2022.912052

**Published:** 2022-08-17

**Authors:** Anna Masek, Anna Kosmalska

**Affiliations:** Faculty of Chemistry, Institute of Polymer and Dye Technology, Lodz University of Technology, Łódź, Poland

**Keywords:** bacterial and plant cellulose, nanocomposites, polymer, technology, biomaterials, biotechnology

## Abstract

Among the many possible types of polymer composite materials, the most important are nanocomposites and biocomposites, which have received tremendous attention in recent years due to their unique properties. The fundamental benefits of using biocomposites as alternative materials to “petroleum-based” products are certainly shaping current development trends and setting directions for future research and applications of polymer composites. A dynamic growth of the production and sale of biocomposites is observed in the global market, which results not only from the growing interest and demand for this type of materials, but also due to the fact that for the developed and modified, thus improved materials, the area of their application is constantly expanding. Already today, polymer composites with plant raw materials are used in various sectors of the economy. In particular, this concerns the automotive and construction industries, as well as widely understood packaging. Bacterial cellulose, for example, also known as bionanocellulose, as a natural polymer with specific and unique properties, has been used extensively,primarily in numerous medical applications. Intensive research is also being carried out into composites with natural fibres composed mainly of organic compounds such as cellulose, hemicellulose and lignin. However, three aspects seem to be associated with the popularisation of biopolymers: performance, processing and cost. This article provides a brief overview of the topic under discussion. What can be the technological limitations considering the methods of obtaining polymer composites with the use of plant filler and the influence on their properties? What properties of cellulose constitute an important issue from the point of view of its applicability in polymers, in the context of compatibility with the polymer matrix and processability? What can be the ways of changing these properties through modifications, which may be crucial from the point of view of the development directions of biopolymers and bioplastics, whose further new applications will be related, among others, to the enhancement of properties? There still seems to be considerable potential to improve the cellulose material composites being produced, as well as to improve the efficiency of their manufacturing. Nevertheless, the material still needs to be well optimized before it can replace conventional materials at the industrial level in the near future. Typically, various studies discuss their comparison in terms of production, properties and highly demanding applications of plant or bacterial nanocellulose. Usually, aspects of each are described separately in the literature. In the present review, several important data are gathered in one place, providing a basis for comparing the types of cellulose described. On the one hand, this comparison aims to demonstrate the advantage of bacterial cellulose over plant cellulose, due to environmental protection and its unique properties. On the other hand, it aims to prepare a more comprehensive point of view that can objectively help in deciding which cellulosic raw material may be more suitable for a particular purpose, bacterial cellulose or plant cellulose.

## Introduction

It should be underlined at the outset that many polymers can be functionalized, making polymers versatile materials. As a diverse material with tunable properties, cellulose can be applied in a wide variety of systems, leading to cellulose-based biomaterials that have some significant benefits over conventional synthetic materials, and being very promising for advancing scientific knowledge.

Small differences in structure can have macro-scale effects. In this context, bacterial cellulose, chemically the same as the well-known plant cellulose, due to its unique nanostructure produced by bacteria exhibits specific and distinct properties that are relevant for tissue function and thus crucial for tissue engineering (Hickey and Pelling, 2019; Ates et al., 2020a; Trache et al., 2020; Seddiqi et al., 2021). In this review, we highlight the importance of nanostructured cellulose-based biomaterials, among others, in several key applications for fundamental scientific research and biomedical engineering.

Growing environmental awareness requires the development of environmentally friendly materials and the reduction of harmful chemicals used in polymer processing. Along with bacterial cellulose, plant cellulose as an inexpensive polymer, hydrophilic, easy to modify, biodegradable and socially acceptable has become an attractive substitute for plastics (Manivel et al., 2021; Omran et al., 2021; Venkatarajan and Athijayamani, 2021). Its further development in polymer composites will be related to improvements in properties, availability and price, the introduction of organic waste collection systems for composting, as well as technological possibilities and limitations in terms of compatibility with the polymer matrix and processability. Thus, in the following chapters we have also focused on current development trends and applications of polymer composites with plant-based cellulosic materials, with particular emphasis on their potential use in the packaging industry, currently considered the largest consumer sector.

Evidently, cellulose-based materials have great potential to become the next generation of standard biomaterials. We stress that the potential uses of these materials are not restricted to the categories reviewed here. We expect that research on cellulose-based materials will continue to grow due to the diversity and versatility of the properties emphasized in this review.

## Bacterial cellulose

Bacterial cellulose (BC), also known as bionanocellulose (BNC), is a natural polymer that, due to its unique properties, has attracted particular attention in recent years from scientists in various fields of knowledge and technology, as well as increasingly from ordinary consumers. Bacterial cellulose (BC) is a biopolymer produced by non-pathogenic bacteria that occur naturally in the environment. It is produced by many different microorganisms, among which bacteria of the genus *Komagataeibacter* (formerly: *Gluconacetobacter*), *Rhizobium*, *Agrobacterium* and *Sarcina* predominate. It is a protective substance for the microorganisms that produce it and, at the same time, an element of the so-called biofilm, which forms a shield against adverse external factors in the form of a mechanically stable network of nanofibers at the interface between an aqueous culture medium containing nutrients and air (Reshmy et al., 2020).

The special properties of this natural nanomaterial result from its unique molecular structure, which is chemically ultra-pure β-1,4-glucan. Chemically, it is the same as the well-known plant cellulose. However, bacterial cellulose bacterial cellulose has a flat ribbon structure and it is precisely because of its unique nanostructure that cellulose produced by bacteria has much more interesting properties than plant cellulose. The chemical structure is the determining factor for the main characteristics of this bionanopolymer, such as high hygroscopicity, flexibility and mechanical strength, which at the same time determine the wide commercial application of BNC (Lin et al., 2011; Olędzki and Walaszczyk, 2020).

It turns out that bacterial cellulose has many more advantages. It is exceptionally pure and is not accompanied by any other substances, such as lignins or hemicelluloses, which are characteristic of plant cellulose. This simplifies the production process as it eliminates the need for purification, which is extremely beneficial from an environmental perspective. It is also significant that the production process of bacterial cellulose is not highly complicated. Only basic bacterial culture conditions are required. The possibility of using cheap raw materials, even waste materials, to produce bacterial cellulose and the biodegradability of the material are another favourable factors. Due to its high hydrophilicity, lack of cytoxicity, biocompatibility and stability over a wide range of temperatures and pH, BNC has been used extensively primarily in numerous medical applications (Halib et al., 2019). The following sections provide a brief overview of the most relevant of them.

## Biomedical applications

### Wound dressings

Research towards applications of bacterial cellulose has been carried out in many directions, as the material can be used in a wide range of industries. Its usefulness in the food, textile and paper industries has been considered but the greatest interest arises in its use in medicine. In the context of medicine, the most important feature of this biopolymer is its biocompatibility, which means that it is non-toxic to human cells and does not cause allergic reactions in contact with the skin (Basu et al., 2018; Hou et al., 2018; Piasecka-Zelga et al., 2018).

**FIGURE 1:**
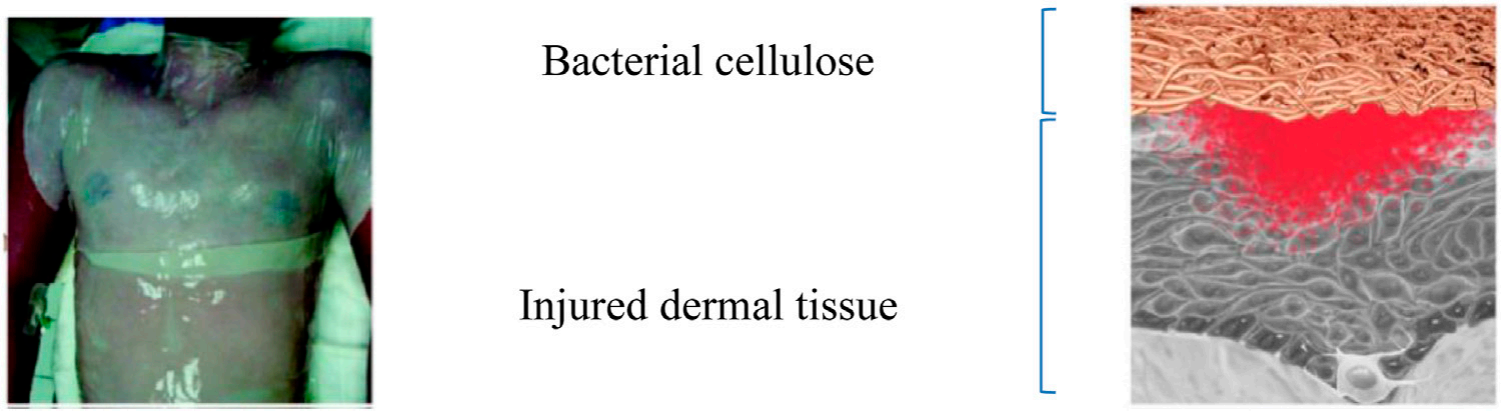
The BC membrane as a wound dressing and the BC network covering the injured area in detail. Source: adapted from (Oliveira Barud et al., 2016).

Cellulose is the first material that meets the requirements for modern dressings to be produced by biotechnology, using the acetic acid bacteria *Gluconacetobacter xylinum*. Human skin does not have self-healing properties for burn wounds. The usefulness of the dressing in wound treatment is therefore being determined. On the basis of preliminary observations, doctors have established that burn wounds protected with cellulose membrane are well insulated from the environment. Nanofibres of bacterial cellulose aggregate and form a hydrated gelatinous membrane (Figure 1). Its hydrated gelatinous structure has a soothing effect in contact with the skin and increases the comfort of patients during dressing changes (Ciecholewska-Juśko et al., 2021). The dressing reduces pain, accelerates skinning in shallow burns and shortens the necrosis demarcation period in deep burns. Such a dressing is characterised by high purity, elasticity and strength. Moreover, its structure allows to mimic the architecture of the extracellular matrix or tissue/organs. Nanofibrillar cellulose hydrogel is a novel material for controlling excessive wound contraction *in vivo* and *in vitro* (Nuutila et al., 2018).

In addition, it is a porous material like a sponge, which ensures gas exchange between the wound and the external environment. In colloquial terms, the wound can “breathe”. Therefore, it is permeable to gases, which prevents the growth of anaerobic bacteria in the places it protects. At the same time, the material is impermeable to microorganisms from outside and protects the wound from secondary infection. The dressing is free of bacterial cells that could cause inflammation (Henschen et al., 2016).

The cellulose dressing also protects against loss of body fluids by securing skinless body surfaces. This is of great importance for patients being treated for extensive burns. The rapid loss of fluid in this type of injury is the cause of frequent patient deaths. Then, the correct division of fibroblasts also does not lead to hypertrophic scars after the wound has healed. Bacterial cellulose has a very high water retention capacity (Wutticharoenmongkol et al., 2019). The strong hydration is a great advantage of the dressing made of this cellulose. Moisture significantly speeds up wound healing. It creates a cooling effect, which is very important for the comfort and pain relief of the burned person. The strong hydration of the cellulose membrane makes it resemble a swollen skin with a very smooth surface, which is why it has been given the terms “artificial skin” or “water jacket”.

It is highly beneficial for the wound healing process to make drug-loaded bandages. Cellulose can be impregnated with antibiotics, enzymes or other substances (Fan et al., 2019). Various materials have also been introduced to develop BC-based biomaterials suitable for wound dressing and with improved properties. Examples in this regard include, e.g., montmorillonitereinforced BC composites, which have been developed as wound dressing and regeneration materials for therapeutic applications without any side effects (Ul-Islam et al., 2013). Another example for wound dressing may be BC-chitosan membranes, which exhibit antibacterial activity and advantageously low cytocompatibility for wound dressings (Lin et al., 2013). Oxidized BC is another kind of cellulose material that is suitable for wound healing because it has significant water absorption capacity, antibacterial activity, and well-dispersed cellulose fibers (Wu et al., 2018a).

Additionally, the cellulosic dressings can be made into any shape and size. The high elasticity of the material allows the wounds to be treated in “awkward” areas, such as the groin or interdigital spaces of the hand.

### Implantology medicine

Due to high mechanical strength, plasticity and flexibility, bacterial cellulose can also be used in implantology medicine–as implants for cartilage, ear, nose or blood vessel. A promising direction in the development of medical engineering seems to be the possibility of using bacterial cellulose tubes especially for the prosthesis of blood vessels with a small crosssection.

The usefulness of the modified bacterial cellulose as a material for tracheal prosthesis was also assessed. Tracheal defects were treated with modified bacterial cellulose with properties corresponding to the cartilage tissue of the organ supplied. Cellulose can also be used as a material for prosthetic intervertebral discs or nasal septal prostheses. Intervertebral disc cartilages, the shape of the auricle or even breast prostheses are modeled from the modified microbial cellulose (Feldmann et al., 2013; Nimeskern et al., 2013; Kolodziejczyk et al., 2017; Apelgren et al., 2021; Khan et al., 2021).

The use of cellulose in the laparoscopic surgical treatment of abdominal hernias has also been noted (Lai et al., 2018). Cellulose fibres are woven into different types of mesh, which allows it to be placed in a laparoscopic tube. The meshwork inserted in this way into the abdominal cavity can replace those used so far, the cost of which is very high.

Using bacterial cellulose is not only much cheaper, but also much safer for the patient. One of the most important characteristics of bacterial cellulose is that which makes it a biocompatible material with a very high level of biocompatibility. The human body reacts differently to an implanted foreign body, often rejecting it or reacting allergically. Cellulose, obtained from bacteria by microbiological synthesis, is very well tolerated by the organism; it even builds into body tissues, which does not modify it in any way. It also excludes the formation of serous cells, which means that the body does not cut off itself from the foreign body and does not surround it with serous fluid, which would then become a potential focus of infection. Another advantage is the incomparably lower risk of transmitting various diseases than with meshworks of animal origin or from donor skin (Gorgieva, 2020).

A review of recent literature indicates great potential for the use of bacterial cellulose for the preparation of tubular biocellulosic materials, with a particular focus on abdominal oncologic surgery. The medical need for the use of biocellulose in modern abdominal surgery is great. Due to its array of properties, biocellulose appears to be a promising candidate for the development of novel soft tissue implants. There are several organ systems that could benefit significantly from the chemical and physical properties of this natural polimer (Bodin et al., 2010; Maia et al., 2018; Klemm et al., 2021). Consisting of 99% water and 1% cellulose, the hydrogel is distinguished by its high purity and biocompatibility and functional hydroxyl groups. The microporous nanofiber network provides mechanical stability, is suitable for all commonly used suturing procedures in surgery and can be colonized with cells. The implant materials can be sterilized and therefore also show stability during long-term storage.

The cited work (Klemm et al., 2021) presents a critical evaluation of the use of biocellulosic materials in terms of the incidence of malignant tumours and surgical interventions and the rate of complications. Using the very first data from large animal experiments as an example, special attention was paid to the regeneration of damaged bile ducts through the use of biocellulose implants. The data obtained may point to biocellulose as a potential candidate for the repair or replacement of abdominal hollow organs. It is important to emphasize that, according to the authors of the review, no definitive studies evaluating the performance of devices have been conducted to date, so such studies assessing the performance of biocellulose-based devices in surgery are indispensable (Klemm et al., 2021).

The potential applications of BC in modern abdominal sugery, especially tendered for hollow organs, are selected in Table 1. This material is particularly recommended for reconstructive parts after excision of a certain part of an organ. Depending on the type of implantation (e.g., interposition or sheath), it may be possible to use the biocellulose as a temporary graft, allowing the organ in question to regenerate along the graft as the lead structure. This would allow removal of the biocellulose after some time. Another approach is to cover a critical area, such as a complex anastomosis, with biocellulose. In most cases, removal of the graft is not possible, so incorporation would be a necessary requirement.

**TABLE 1:** A clinician’s perspective on potential applications of biocellulose in modern abdominal surgery.

Organ	Potential for tubular cellulosis application	
Bile ducts	For reconstruction of the biliary anatomy, an interposition is possible and from great clinical value	High
Ureter/Urethra	For reconstruction of the ureter/urethal anatomy, an interposition is possible and from great clinical value	
Esophagus	Either as interposition after esophageal resection or as sheathing of an anastomosis	
Large intestine	Conventional methods for reconstruction even after removal of larger parts of the large intestine established; Sheathing of an anastomosis after rectum resection could be an application	Medium
Pancreas	Sheathing of an anastomosis after pancreatic head resection could be an application	
Small intestine	Conventional methods for reconstruction even after removal of larger parts of the small intestine established	Low
Stomach	Conventional methods for reconstruction after (partial) gastrectomy established	

### Scaffolds for tissue engineering

Scaffolds, known as artificial extracellular matrices, are three-dimensional structures whose essence is to participate in the reconstruction of a diseased or damaged organ. The purpose of the artificial matrix is to act as a rigid scaffold onto which living cells can be seeded, so the material should be constructed in such a way that viable cells can be grown in it.

The artificial matrix enables cells to differentiate, proliferate and maintain normal metabolic and catabolic processes due to the fact that its structure is similar to that of naturally occurring cell matrices. The idea is to place the scaffolds inhabited by living cells in the body of a sick patient, in the place of a damaged or diseased organ/tissue, where it will come to its regeneration. Once completed, the scaffold should degrade and adsorb to tissue, eliminating the need for surgical removal (Chan and Leong, 2008; Ashjaran, 2013). Scaffolds can be made of different types of polymers, natural or synthetic.

Although many methods of obtaining scaffolding are known, most of the materials used in their production are not biodegradable. Some of the methods are also complicated and very expensive (Ashjaran, 2013; Kaźmierczak et al., 2016). Bacterial cellulose has all the properties that scaffolds should have (Wang et al., 2011; Kaźmierczak et al., 2016). It is very important to point out here that the bacterial nanocellulose network has a very high affinity for water, which results in its hydrogel-like properties and provides an ideal environment for host cells (Petersen and Gatenholm, 2011). Furthermore, BC production costs are low. The only drawback is that the channels and chambers are too small to grow living cells in, as the natural pores present in the cellulose structure do not have adequate diameters for viable cellls colonization. It is very important to emphasize that organ reconstruction using scaffolds would help significantly in solving the problems of transplantology. For example, in Poland in 2016, only 7.3% of people waiting for a transplant got a chance to recover.

A very promising technique involves repeated frosting and defrosting of cellulose samples in order to ovecome the problem. During the proces, i.e. freezing and thawing, the volume of freezing water increased by about 10% each time. This phenomenon caused disruption of the cellulose structure. Kaźmierczak (2018) conducted some experiments aimed at enlarging the channels in the cellulose structure by using this technique.

Multiple frosting and defrosting combined with application of a sterile mixture of vegetable oil in the cultivation process yielded successful results. Studies have shown that only then the diameter of the channels and chambers was large enough to be colonized with living cells. According to the authors of the studies, it is worth noting that all of the experiments were successfull, as freezing and immersion the cellulose membranes in boiling water induced greater pores but not large enough to provide the necessary support for various cellular functions. The oil-ethanol mixture applied on the membrane during its formation entailed more pores in the cellulose structure. Ultimately, it was the application of the oil that resulted in obtaining properties, which theoretically allow the scaffolds to comply with all conditions. Other researchers (Oliveira Barud et al., 2015) have proposed another technique, namely freeze-drying, for obtaining bacterial-cellulose sponge scaffolds with the addition of silk fibroin (SF). According to the authors, the SEM images they obtained showed a higher number of fibroblast cells attached to the surface of the BC/SF:50% scaffold compared to the surface of pure BC. This may indicate that the presence of fibroin improves cell adhesion, which may be related to the amino acid sequence of SF, which acts as cell receptors to facilitate cell adhesion and growth. Thus, bacterial cellulose scaffolds combined with silk fibroin are an excellent option in bioengineering, indicating their potential in tissue regeneration and cell culture on nanocomposites. Cell adhesion to substrate surfaces in cellulosic materials can also be improved by other means, such as the addition of matrix ligands. To adsorb collagen on membrane surfaces, which can promote cell adhesion, ionic charges can be added to cellulose membranes, for example (Courtenay et al., 2017).

The same technique of freeze-drying method has been applied to other studies on the biology of adipose tissue and metabolic diseases, which are currently of increasing interest. For this purpose, Krontiras et al. (2015) prepared 2D and 3D porous scaffolds by crosslinking homogenized cellulose fibers with alginate and then freeze-drying the mixture to obtain a porous structure. The authors concluded that 3D culturing of adipocytes in macroporous BC scaffolds is a promising method to generate adipose tissue as an *in vitro* model, as the cells cultured in 3D macroporous scaffolds contained more cells growing in clusters with large lipid droplets compared to 2D scaffolds. Moreover, they also proposed that BC-alginate could be used as an injectable gel to enable the delivery of fat cells directly to the defects to be repaired. Other examples of combining bacterial cellulose with additional materials for tissue engineering purposes include, for example, graphene oxide. Graphene oxide and bacterial cellulose (GO/BC) nanocomposite hydrogels were prepared by *in situ* biosynthesis method by adding graphene oxide suspension to BC culture medium, resulting in well-dispersed GO in BC network (Luo et al., 2014; Si et al., 2014). Such a result obtained imparts the positive impact on efficient load transfer between reinforcement and matrix by improving by about 38 and 120% the tensile strength and Young’s modulus of the material, respectively.

As can be seen from the above examples, the appropriate mechanical properties of biomedical materials are obviously crucial and very specific to the application area. For example, the elastic modulus of the material must be close to the tissue that the material replaces or strengthens. In this sense, nanocrystalline cellulose is a promising material for cell attachment and proliferation not only because of its biocompatibility but also its excellent mechanical properties. In addition, such a network can be further mechanically strengthened, for example by cross-linking individual nanofibers.

As shown, the improvement of scaffold properties for tissue engineering can also be achieved by using a suitable reinforcing material, such as graphene oxide, as proposed by Si et al. (2014) and Luo et al. (2014), already quoted above. Another example is a more complex hybrid scaffold based on bacterial cellulose synthesized by bacteria in a medium containing carbon nanotubes (CNTs) and additionally coated with an amphiphilic comb polymer (Park et al., 2015). The scaffolds so obtained exhibited excellent osteoconductivity and osteoinductivity, providing high bone regeneration efficiency. According to the authors, it may also be a suitable material for developing biofunctional 3D scaffolds for regenerative medicine.

But there could be more examples. Indeed, in the literature one can find many biomaterials that have been associated with BC, such as collagen, gelatin, fibroin, propolis, chitosan, silver, alginate, hydroxyapatite, montmorillonites, BC nanowires for reinforcing materials, and others (Jinga et al., 2014; Sajjad et al., 2019). Some of the materials mentioned, such as collagen and silk fibroin (Xiao et al., 2014; Kim et al., 2018), have been studied for corneal transplantation, which, despite achieving considerable clinical success, still have several limitations due to specific issues. Among the most important are corneal tissue deficiency, graft failure, or immune rejection, etc. In the last 10 years, significant advances have been made in this field, including corneal tissue engineering, which overcomes the problem of tissue shortage. However, the aforementioned materials have been shown to have difficulty meeting requirements for corneal regeneration, in particular in terms of transparency, water and nutrient permeability, mechanical strength and stability. In contrast to these materials, bacterial cellulose (BC) is proving to be a very promising material as a potential corneal scaffold, as shown by some of the current literature on the subject (Picheth et al., 2017; Zhang et al., 2020). In addition to being distinguished by its high light transmittance, it exhibits favorable mechanical properties, making it capable of resisting surgical sutures and intraocular pressure.

Besides these important issues in terms of mechanical strength, the rate of degradation of the scaffold under given conditions is also usually an important consideration. To ensure that the damaged tissue is completely replaced by healthy tissue and to restore its function, it is assumed that the degradation rate of the scaffold should correspond to the time of tissue formation.

### Other applications

Various physical and chemical forms of bionanocellulose have found applications in food processing, such as a stabilizing and preservative food additive, and as an agent to improve the properties of semi-liquid products (Martins et al., 2018; Azeredo et al., 2019a), as a bulking, thickening, texturizing, and calorie-reducing agent (Mesomya et al., 2006), or as a food shelf life extender (Sabularse et al., 2009; Suppakul et al., 2010).

Currently, attempts are also being made to produce packaging from bacterial cellulose for food products. Packaging based on biological materials is becoming a great hope for the packaging industry due to the low cost of production and their low environmental impact. Due to the fact that BC packaging is fully biodegradable, these solutions provide an opportunity for their dissemination and mass use in industry and household (Sonia and Dasan, 2013; Arrieta et al., 2014; Shi et al., 2014). Furthermore, it has been shown that the oxidation process, in which the hydroxyl groups of cellulose are oxidized to aldehyde, ketone or carboxyl groups, results in bacterial cellulose acquiring antimicrobial properties. For this reason, oxidized BC can be a potential ingredient in packaging to extend the microbiological shelf life of the packaged food product (Tabaii and Emtiazi, 2016).

Other applications for bacterial cellulose include loudspeaker membranes, electrical wire insulation, artwork reconstruction, air filters, or ultra-durable paper. The possibilities are endless. Interesting possibilities arise as a result of obtaining nanofibers from bionanocellulose, produced by electrospinning. The unique properties of the nonwoven fabric such as porosity (92–94%), large surface area and dense cross-linking of polymeric threads enable its use both in medicine and industry.

However, despite intensive research, the production costs of this biopolymer are still considered high and the process efficiency unsatisfactory on an industrial scale. The relatively low efficiency of biotechnological processes in industry is the reason for the growing interest in the application of specific types of magnetic fields (MFs). A considerable number of current literature items indicate their use to stimulate the growth rate of microorganisms and change their metabolic activity in order to obtain a satisfactory yield of secondary metabolites, significant from industrial point of view (Buchachenko and Lawler, 2017; Rekena et al., 2019; Drozd et al., 2021). There is still significant potential to improve manufactured biopolymer materials and improve the efficiency of their production.

## Plant cellulose

Cellulose, considered the most widespread organic material and polysaccharide on Earth, occurs in nature primarily as microfibrils in the cell walls of wood and plants, algal cell wall, and the Ascidian sac of tunicates. Traditionally, cellulosic materials have been used in industry to make paper and textiles.

All the specific properties of cellulose make it an attractive substitute for plastics. As a replacement for traditionally used synthetic materials, it is expected to show similarly promising performance characteristics while maintaining an acceptable level of efficiency. Cellulose fibres of wood or plant origin have long shown potential as a reinforcement for composites (Ramamoorthy et al., 2015), in addition to the commonly used glass fibres (Thomason, 2019) and carbon fibres (Chawla, 2013). The challenge is to find a biobased option for the composite matrix that is responsible for fibre binding, as two chemically different components often have poor interfacial compatibility, which can lead to water absorption, reduced mechanical properties of the material (Nishino and Arimoto, 2007) and shorter product life. In the following sections, a brief overview of the most relevant applications of plant cellulosic fibres in the field of polymer bio-composites is presented, taking into account the technological possibilities and limitations of cellulose processing, and with a special focus on the applicability of this biomaterial in the packaging industry, currently considered as the largest consumer sector.

### Packaging applications

The plastics market is an important part of the global economy. For example in Poland, the demand for plastics is 3.5 million tonnes per year and the biggest consumer is the packaging sector. This sector consumes 30–40% of all plastics processed. The biggest role is played by polyolefins: LDPE and LLDPE, PP and HDPE, as well as PET. These polymers account for more than 80% of the plastics demand for packaging. On the other hand, 26% of the plastics produced are used in the construction industry and one tonne in ten is used for the automotive industry.

There are significant differences in the lifespan of different plastic products. Some are used for a very short period of time while others last for decades. However, the problem arises when we no longer need a given product or plastic packaging. When thinking about polymer technology, a great challenge is to minimize plastic waste (Datta and Kopczyńska, 2016; Liu et al., 2019) and to reduce environmental pollution (Platnieks et al., 2020).

Growing environmental awareness requires the development of materials that are less harmful to our surroundings and make use of the natural environment around us without harm. In order to achieve a compromise between sustainability principles and polymer technology, it is very important to use environmentally friendly materials (Barczewski et al., 2019a; Bartos et al., 2020; Ates et al., 2020b) and to reduce harmful chemicals used in polymer processing (Lisuzzo et al., 2019; Yang et al., 2019; Bertolino et al., 2020). Therefore, the search for natural substitutes is a current need.

Biodegradable and compostable packaging made of bioplastics is therefore today an example of an ecological alternative to plastic films and fits perfectly into the principles of the ClosedLoop Economy. Cellulose is a polymer that is cheap, hydrophilic, chiral and easy to modify chemically. It is biodegradable and socially acceptable. All these characteristics make cellulose an attractive substitute for plastics. However, despite these undoubted advantages and indisputable arguments, bioplastics are still at an early stage of development and occupy a small market niche. Their further development will be linked to improvements in properties, availability and price, as well as the introduction of organic waste collection systems for composting.

Technological possibilities and limitations are also not insignificant. To analyze this issue, it is worth looking at the properties of cellulose, which may determine the possibilities of its use in polymer processing. An important aspect in this regard is, first of all, the question of compatibility of cellulose fibers with the polymer matrix. The hydrophilic nature of cellulose contributes to the widespread use of water-soluble matrices in the production of composites. However, the use of nanofibers and cellulose nanocrystals in composites with hydrophobic matrices may encounter problems due to weak interfacial bonding.

Thus, the moisture content of cellulose fibers is an extremely important parameter, as it determines a number of its properties. The ability to absorb water influences the molecular packing, the stresses occurring inside the fibers, the mobility of the polymer chains or the availability of active centers important during modification, as well as the size of the pores. Cellulose in equilibrium with the atmosphere always contains absorbed moisture, generally between 4 and 5% by weight. (Mihranyan et al., 2004). Considering the applications in polymer composites where fibers act as a filler, this may be a certain disadvantage and limitation. This is because the water content may contribute to poor mechanical properties of the polymer composite (Espert et al., 2004), poor adhesion of the filler to the hydrophobic polymer matrix (Chen et al., 2009) and result in a decrease in the decomposition temperature, i.e., a deterioration in the thermal stability of the material (Sombatsompop and Chaochanchaikul, 2004). Moreover, according to the available information, it can be concluded that water content significantly contributes to cellulose degradation. The hydrolysis reaction of the glucosidic bonds contributes to the attachment of unstable acetal chain ends and to the reduction of the molecular weight. Moreover, considering that water is the main product of the thermal decomposition of cellulose, especially during the initial phase of thermal treatment, the considered process can be attributed to autocatalytic reactions (Scheirs et al., 2001; Poletto et al., 2012; Poletto et al., 2014). In this situation, the moisture content of the biopolymer is of great importance to obtain a material with the best possible properties (Manaf et al., 2011; Liu et al., 2017; Leszczyńska et al., 2019; Mao et al., 2019).

According to the literature, the changes that the water contained in cellulose undergoes during conventional heat treatment can be divided into the following three stages (Scheirs et al., 2001): physical water loss (<220°C), chemical water loss (220–550°C), and chemical water loss in pyrolysis (>600°C). From the point of view of materials science, the first and second stages of water loss are the most important. The first of these relates to the problem of drying cellulose prior to incorporation into a polymer matrix. Then, the subsequent chemical water loss process (220–550°C) can help to understand and analyze the thermal behavior of the produced polymer composites with respect to the initial moisture content of the cellulose fibers.

Our research in the field of hydrophobization of cellulose fibers, conducted for potential applications to polymer composites, includes the replacement of heat treatment with a new method (Cichosz and Masek, 2019). Although published studies concern cellulose fibers (Arbocel UFC100 - Ultra Fine Cellulose), but considering its chemical structure and that of bionanocellulose, it is the same. Thus, any modifications can be carried out very similarly, which may be important from the point of view of the directions of development of biopolymers and bioplastics, whose further development and new applications will be related, among others, to the improvement of properties.

**FIGURE 2:**
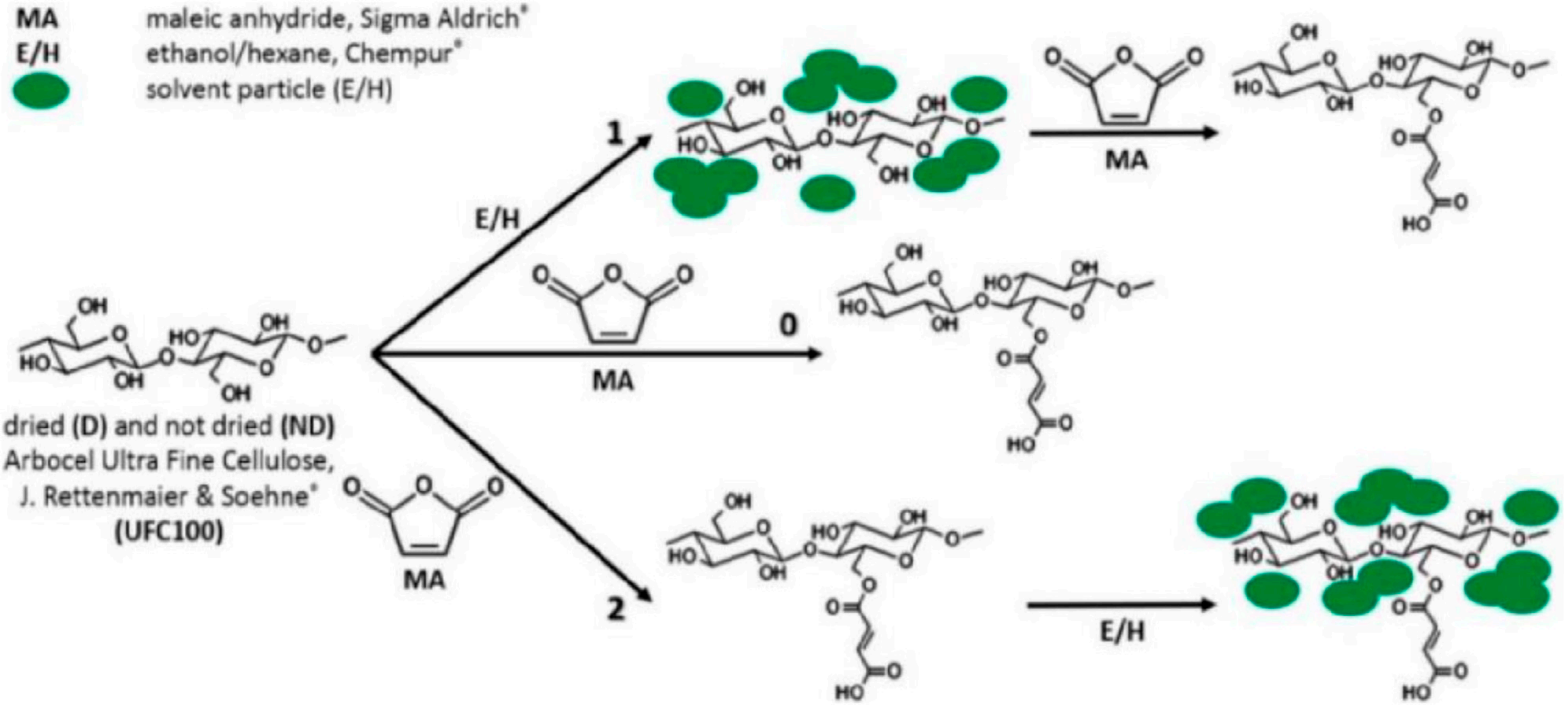
Hybrid chemical modification of cellulosic fibers: pathway 0-regular surface modification with maleic anhydride (MA); pathway 1-solvent exchange before surface modification with MA; path 2-solvent exchange after surface modification with MA (Cichosz and Masek, 2019).

In a published study, we proposed and described a novel hybrid chemical modification method using maleic anhydride and solvents of different polarity to minimize the moisture content and intensify the cohesion forces of the filler-matrix pair (Figure 2). This is the reverse of the commonly used process of grafting the polymer matrix with maleic anhydride (MA) followed by blending with cellulose. In our work, we presented an alternative approach that aims to use MA not as a direct coupling agent (i.e., coupling agent) of the cellulose to the polymer matrix, but as an agent that changes the surface properties of the cellulose (e.g., hydrophobicity, specific surface area).

Thus, the described method consists of two steps: solvent exchange (water to ethanol or hexane) and chemical modification by maleic anhydride (MA) grafting. Previously, the concept of solvent exchange was proposed by Ishii and coworkers (Ishii et al., 2003). It was found then that the presence of solvent molecules between cellulose macromolecules relaxes the surface fractal of microfibril aggregates. As a consequence, the aggregate geometry changes to a bulk fractal (Laine et al., 2003). The overall conclusion of the study by Ishii et al. is that solvent exchange improves molecular mobility and shortens the characteristic length along cellulose microfibrils (Ishii et al., 2003). We therefore used the chemical filler modification process implemented in our study to alter the molecular mobility of the biopolymer during solvent exchange. As a result, the cellulose underwent changes in physical structure (swelling in solvent) and surface properties (by chemical grafting with maleic anhydride MA) after complex treatment.

By means of infrared spectroscopy (FT-IR), it has been demonstrated that the use of different solvents can contribute to the efficiency of the modification process, as they cause rearrangements in the hydrogen bond structure, as well as swelling of the biopolymer, consequently affecting its molecular packing. Based on the results obtained, it can be concluded that the use of ethanol greatly contributed to the reduction of water absorption capacity of cellulose (Cichosz and Masek, 2019).

Moreover, investigations carried out using thermogravimetric analysis (TGA) and differential scanning calorimetry (DSC) revealed an improvement in the thermal resistance of the fibers as a result of the new hybrid chemical modification. A shift in the value of 5% temperature loss in weight from 240 to 306°C was observed as a result of the use of a solvent in the modification process.

We extended the study to analyze the effect of cellulose moisture content on the modification process by drying the test fibers, or not, prior to hybrid chemical modification. Based on the results obtained, we found that cellulose pre-dried before modification showed increased heat resistance, while non-dried fibers were more susceptible to maleic anhydride (MA) modification (Cichosz and Masek, 2019).

It should be emphasized that for all modifications carried out, a reduction in moisture content was observed, ranging from about 4% for thermal drying to 1.7% for hybrid MA modification. This result is extremely promising considering the possibility of using the treated fibers in a polymer matrix. It is also worth noting that MA treatment can contribute to the formation of nanofibrils (Iwamoto and Endo, 2015).

We found the presented experimental results to be a promising and effective way to improve the interactions of the cellulose filler with the potential polymer matrix. Therefore, the next research was concerned with the application of modified fibers in the polymer.

As a result, we obtained and described the properties of composites based on ethylenenorbornene copolymer (TOPAS Elastomer E-140) filled with cellulosic plant fibers (Cichosz et al., 2020). It is worth noting that similar systems, e.g., polyethylene (PE) or polypropylene (PP) modified with MA, similar to ethylene-norbornene copolymer filled with natural fibers, have been reported in the literature as polymer composites with good mechanical characteristics (Chen et al., 2013; Li et al., 2014; Yeh et al., 2014; Sato et al., 2016).

Therefore, we evaluated the change in the filler structure and stiffness of the polymer composite based on ethylene-norbornene copolymer obtained with its participation, as well as the tensile strength and elongation at break using static mechanical analysis methods. As a result, the conducted tests showed a significant improvement in the performance of the composite, tensile strength of 38.8 MPa and 510% elongation at break (Cichosz et al., 2020). The values obtained are higher than for the pure polymer matrix and, importantly, previously impossible to achieve as a result of regular modification with maleic anhydride (MA). Considering the available literature data, for example, in the case of a polyethylene-based composite filled with modified cellulose fibers, which is a system similar to ethylene-norbornene copolymer, the tensile strength usually varies between 25 and 40 MPa (Arrakhiz et al., 2012; Sato et al., 2016), depending on the source and type of polyethylene.

Dynamic Mechanical Analysis (DMA) can provide valuable information regarding rheological properties such as viscosity, storage modulus and loss modulus data, and Payne effect (Essabir et al., 2013; Saba et al., 2017; Barczewski et al., 2019b). In the case of our study, it was carried out to evaluate the reinforcing nature of the biofiller and to confirm the results of the static tensile test.

In order to understand the changes in polymer composites occurring due to the reinforcing effect of fillers, many theories have been proposed to explain the Payne effect, e.g., clustering of particles in the network in the form of clusters or through physically stuck domains due to filler-matrix interfacial interactions (Robertson and Wang, 2005). It should be noted here that a well-developed filler structure does not always correlate well with improved performance of the composite, as the tendency of the filler to aggregate can be misunderstood in terms of its correct dispersion within the polymer matrix (Jordan et al., 2005). Therefore, an additional factor was established to estimate the behavior of the filler in the polymer matrix, namely the filler volume fraction, which compares the storage modulus of the unfilled and filled system with respect to the volume fraction of filler in the matrix. The study described (Cichosz et al., 2020) shows that both the Payne effect and the cellulose filler capacity factor, although not in all samples considered, indicate the possibility of a reinforcing nature of the fibers, which is not a common result.

Summarizing at this stage, it should be emphasized that the effect of polymer matrix strengthening is a very complex phenomenon. Apart from the fact that it depends on good distribution of the filler and its structure in the polymer matrix, other factors, e.g., filler-polymer matrix adhesion, properties at the phase boundary, as well as possible entanglements, are also of great importance (Gurovich et al., 2011).

What should be emphasized is that the observed strengthening effect, according to the literature, is a general resultant of two different mechanisms occurring simultaneously; the strengthening effect of the polymer matrix by the filler, as well as the result of interfacial bonding between cellulose and ethylene-norbornene copolymer (Barczewski et al., 2018).

In addition, the materials described in the cited articles are extremely promising considering the potential use of biopolymer composites in common healthcare applications due to the fact that ethylene-norbornene copolymer is widely used in this field. As a polymer matrix, it has high purity, excellent barrier properties and can be sterilized by all known methods. In addition, it is widely known for its excellent resistance to aqueous and polar organic media, good biocompatibility, and ability to reproduce fine structures, making it an interesting material for medical applications. Furthermore, its ease of fabrication provides molding possibilities that were not available, for example, for glass products (mainly used in the past). Therefore, plant fiber-based polymeric materials described in the cited studies may find potential applications in areas related to medical devices, drug delivery, in the manufacture of trays, pharmaceutical blister packs, or other items in contact with the body. Nevertheless, this material still needs to be well optimized in the future.

### Composites–current development trends in this field

Polymer-matrix composites dominate in terms of application in technology over the other types, i.e. metallic or ceramic matrix composites. Polymer matrix composites are among the materials with the highest strength-to-weight ratio. Thanks to the use of polymers in composite materials they gained lightness, resistance to corrosion, vibration damping ability, electrical and thermal insulation and ease of shaping. They are characterized by very high elasticity and extensibility.

Polymer matrix composites used mainly in the packaging, automotive and medical industries are of the greatest practical importance. The unique properties of polymer composites are appreciated by an increasing number of customers, resulting in their growing use in the aerospace industry (Ghori et al., 2018; Irving and Soutis, 2019). They are also more and more often used for manufacturing of many specialized industrial products, e.g. rolling elements of printing machines, computer and machine housings, medical equipment. They even turned out to be indispensable, e.g., in the production of wind turbine components. In the construction industry, polymer composites have become popular due to their low weight and high strength of structural elements, but also due to the ease of installation, operation, corrosion resistance and the possibility of covering their surfaces with special anti-graffiti paints (Mosallam et al., 2021). The global market for polymer composites is growing rapidly and will also continue to grow in the future not only because of increasing demand from industries that are their main customers, but also because new applications are being found for them.

Among the many possible types of polymeric composite materials, the most important are nanocomposites and biocomposites, which have received great interest in recent years due to their unique properties (Bajwa and Sujal, 2016; González, 2017; Formela et al., 2018; Sun et al., 2018). The attraction in the case of nanocomposites stems from the fact that the polymer matrix and the nanofiller interact already at the molecular level. Thus, the nanofiller, which is less than 100 nm in size, usually added in an amount of a few percent to the matrix can significantly alter selected properties of the composite material. For example, nanocomposites with aluminosilicate fillers have found applications in car engine parts, aerospace, etc. However, ceramic fiber-reinforced composites are considered to be the products of the future in this field. These extremely strong materials are already being tested in aerospace, among others.

In turn, the fundamental benefits of biocomposites as alternative materials to “petroleum-based” products are certainly shaping current development trends and guiding future research and applications of polymer composites. Thus, the prevention of demand-supply imbalance of products made from non-renewable fossil raw materials, sustainable waste management, reduction of carbon dioxide emissions, biodegradability of plastics or facilitated recycling process in the case of biocomposites make polymer composites with plant-based raw materials applicable in various economic sectors, especially in the automotive and construction industries. There is also a dynamic growth in the global market for the production and sale of biocomposites, which is not only due to the growing interest and demand for such materials, but also because for the materials developed and modified, thus improved, the field of application is constantly expanding. Current trends in the development and application of biodegradable composite materials already cover fields such as artificial joints, wound care, delivery of appropriate drugs and orthopedic components to the patient’s body and are also widely used in food packaging and agricultural films (Chieng et al., 2014; Saba et al., 2014; Yusoff et al., 2016).

Polymers, which can degrade naturally, play an important role in solving the problems and reducing the risks of polymeric materials and maintaining the ecological balance. However, there are three aspects associated with the promotion and popularization of biopolymers: performance, processing, and cost. The inherent disadvantages of some biodegradable polymers, such as poor mechanical properties, narrow processing window, and low electrical, thermal, and barrier properties, can be overcome by adding suitable reinforcing fillers using advanced technologies and methods (Chen et al., 2015; Chen et al., 2016a; Sha et al., 2016). Thus, intensive research is being done on composites with natural fibers composed primarily of organic compounds such as cellulose, hemicellulose and lignin. Properties of polymer composites made of plant raw materials depend mainly on phase boundary interactions, which may be difficult in case of hydrophobic matrix and hydrophilic filler. The proper selection of the ratio between the matrix and the used additive is also important. Therefore, especially important for the development of these materials seems to be the continuous search for new, competitive fillers, both inorganic and derived from renewable plant sources, as well as conducting various modifications of them and introducing other additives. Thus, current trends in the development of polymer biocomposites often focus on the development of methods, or means, to improve the compatibility of the composition components (Wei et al., 2015; Gandini and Belgacem, 2016; Vaidya et al., 2016; Lenfeld et al., 2020). Among the most commonly used methods, chemical modification, such as impregnation of fibers with a matrix-compatible polymer, graft copolymerization, acetylation, mercerization, or physical modification, such as corona discharge, thermal or plasma treatment, stand out. These modifications, in addition to improving adhesion, are primarily intended to reduce water absorption and increase the dimensional stability of the product.

The research carried out, apart from the formulation, usually includes the characterization of mechanical, rheological and thermal parameters as well as the structural analysis of composites obtained with plant fillers of various origin. Additionally, the tests are conducted to determine the stability of the obtained materials, subjected to the influence of microorganisms (biodegradation) and environmental factors. An important issue is the development of effective technologies to obtain homogeneous composites with good, often unique functional properties desired for a specific type of application. There is still a significant potential to improve the produced composites as well as to improve the efficiency of their manufacturing.

A particular example that should be mentioned when considering trends in polymer composites development is the obtaining and application of hybrid bio-nanocomposites, consisting of a biopolymer matrix and nanoparticles that are used for reinforcement or functionalization (Pracella et al., 2014; Wu et al., 2018b; Carvalho et al., 2018). Polymer composites that exploit the synergistic effect between the filler and the biodegradable polymer matrix can then both lead to improved properties and meet practical requirements and environmental degradation. The large aspect ratio and large specific surface area of the nanoparticles make bio-nanocomposites a new type of material that has significantly improved functional properties compared to basic biopolymers (Chen et al., 2016b; Xie et al., 2016; Chen et al., 2017). Biodegradable nanocomposites are mainly developed for use in electronics, packaging and biomedical applications. Currently, in electronics, nanocellulose composite materials can be used to fabricate electronic displays, flexible sensors, light emitting diodes, etc. to promote the development of flexible electronic devices. In the field of packaging, nanofiller-enhanced biodegradable composite materials can overcome the aforementioned problems limiting their popularization and help improve the performance of biodegradable polymers.

On the other hand, the use of nanofillers to reinforce biocomposites in medicine is being explored to enhance their functionality and to seek breakthrough applications, which in biomedicine mainly include the fields of tissue engineering, drug delivery and gene therapy. Nanofiller-reinforced biodegradable composite materials are therefore being used more widely and this area is undoubtedly attracting widespread attention and interest (Abitbol et al., 2016; Mishra et al., 2018a; Chin et al., 2018). In this context, it is worth noting that this does not only apply to bacterial cellulose, whose advantages over plant cellulose have determined the predominant medical applications. A literature review of the last 3 years reports, among other things, the use of plant cellulose for bone implants (Osorio et al., 2019). This is a special structure obtained from plant cellulose, with a foam-like structure, which can be injected into the body and thus create a scaffold for new bone growth. The described substance is created by processing nanocrystals obtained from plant cellulose in such a way that they combine into a strong but lightweight sponge. Technically speaking, a kind of aerogel is formed, which can compress or expand as needed to completely fill the defect in the bone cavity.

It is anticipated that in future studies, researchers are likely to focus on nanofillers in composite materials, and to explore more industrialized and efficient processes that are generally difficult to perform with nanotechnology. In the future, biodegradable composite materials could replace most current materials, which could go a long way toward sustaining our lives.

## Comparison of bacterial cellulose and plant cellulose

In this section, several important data, including production methods, properties and applications, are compiled in one place, providing a basis for comparing the types of cellulose described in this study. This comparison shows that both bacterial cellulose and plant nanocellulose have specific properties that will determine the choice of cellulose type for a given application.

According to information gathered from the Science Direct website, the number of publications on both plant nanocellulose and bacterial cellulose is increasing every year and that bacterial cellulose is receiving more attention from researchers. This can be said to be due to its unique properties and high purity, as discussed earlier.

In general, nanofibers and nanocrystals of plant cellulose can be extracted from plants by means of a mechanically or chemically assisted deconstructing process (Postek et al., 2011; Klemm et al., 2018). Given that the release of nanocellulose from plants requires processing to disaggregate the various constituent materials, and that the most common pretreatment steps are milling, pulping and bleaching, the production process for plant nanocellulose can be referred to as “disintegration” (Table 2). Occasionally, one can also encounter in the literature the term “bottom-down” strategies. In turn, given that the synthesis of bacterial cellulose from species of the genus Gluconacetobacter is a complex process involving the polymerization of glucose monomers and the release of cellulose into the external environment, resulting in the formation of a three-dimensional network, one can conceive of the whole process as a “build-up” strategy for a material with a unique structure (Klemm et al., 2005; Mishra et al., 2018b; Phanthong et al., 2018).

**TABLE 2:** Comparison of properties between plant and bacterial cellulose.

Properties	Plant cellulose	Bacterial cellulose	References
Production process	Disintegration	Build-up	Klemm et al. (2005); Postek et al. (2011); Mishra et al. (2018b); Klemm et al. (2018); Phanthong et al. (2018)
Crystallinity degree	54–88% (cellulose nanocrystals)	65–79%	Phanthong et al. (2018)
	59–64% (cellulose nanofibers)		
Particle size; length	0.05–0.5 µm (cellulose nanocrystals)	>1 µm	Pecoraro et al. (2008)
	0,5–2 µm (cellulose nanofibers)		
Particle size; width	3–10 nm (cellulose nanocrystals)	30–50 nm	Pecoraro et al. (2008)
	4–20 nm (cellulose nanofibers)		
Degree of polymerization	500–15,000 (cellulose nanocrystals)	800–10,000	Pecoraro et al. (2008)
	≥500 (cellulose nanofibers)		
Young’s modulus	50–100 GPa (cellulose nanocrystals)	15–30 GPa	Pecoraro et al. (2008)
	39–78 GPa (cellulose nanofibers)		
Purity	Low	High	Pecoraro et al. (2008); Mishra et al. (2018b)
Environmental impact	Production of polluting reactions and environmentally harmful compounds	No undesirable compounds	Donini et al. (2010b)
Limitations	Application of cellulose nanofibers and cellulose nanocrystals in composites with hydrophobic matrices limited by weak interphase interactions	Lack of efficient large-scale fermentation systems; still very incipient commercialization systems	Bharimalia et al. (2017)
			Da Gama and Dourado, (2018); Azeredo et al. (2019b)
			Amorim et al. (2020)
Industrial scale production	Limited	Under research and implementation	Phanthong et al. (2018)
			Da Gama and Dourado, (2018); Azeredo et al. (2019b)
			Amorim et al. (2020)

Considering the issue of composite manufacturing in the context of the important differences between plant cellulose and bacterial cellulose, cellulose nanofibers and nanocrystals have attracted considerable interest as reinforcing agents for developing nanocomposites with polymer matrices (Bharimalia et al., 2017; Hisseine et al., 2019). At the same time, bacterial cellulose has been identified as a matrix for particle deposition of a wide range of reinforcing materials (Hobzova et al., 2018).

In terms of the environmental impact of the production method, bacterial cellulose has an advantage over plant nanocellulose. Obtaining bacterial cellulose has simple upstream and downstream processes with no undesirable compounds (Donini et al., 2010b) and importantly contributes to reducing the cutting of trees needed to produce cellulosic material. In contrast, obtaining plant nanocellulose unfortunately involves the production of environmentally harmful compounds. This is primarily because in the production of plant cellulose, it is necessary to separate cellulose from other compounds (such as hemicellulose and lignin), which occurs through extremely polluting reactions. The chemical purifcation of plant based cellulose involves alkaline extraction and bleaching.

On the other hand, since there are companies already producing kilograms or tons a day it means that plant nanocellulose shows more potential for large-scale production. Despite many efforts, designing a cost-effective process for producing bacterial cellulose and scaling up the culture is still a challenge, as a major limitation is the lack of efficient fermentation systems and the high cost of the traditionally used Hestrin-Schramm culture medium (Da Gama and Dourado, 2018; Azeredo et al., 2019b). Nevertheless, the production costs are also high for the commercialization of plant nanocellulose production. In this case, the following factors should be taken into account: high cost of chemicals, maintenance of equipment operated in an acidic environment, difficult treatment of acidic wastewater, and high energy consumption for the treatment (Phanthong et al., 2018).

The molecular formula of bacterial cellulose is the same as that of plant cellulose, however they differ in morphology, the same as in physical and chemical properties. Plant nanocellulose has a low degree of crystallization when compared to other celluloses obtained from different sources, but when it has a relatively high degree of polymerization, it tends to show an increase in crystallinity and in its mechanical resistance. In terms of structure, bacterial cellulose is characterized by porosity and has more hydroxyl groups on the surface than plant fibers. This individual characteristic can be successfully exploited, for example, in electronic technologies, as it favors doping with conductive materials such as carbon nanotubes, graphene oxides or also metal nanoparticles (Ma et al., 2016). In contrast, the lack of pores, lower number of hydroxyl groups and low mechanical resistance in the case of plant nanocellulose prevents the incorporation of nanowires (Balea et al., 2019).

With regard to plant nanocellulose, a morphological differentiation can be observed between nanocrystals and nanofibers. Cellulose nanofibers are fibrillar in nature (Liu et al., 2014), while cellulose nanocrystals are stalk-shaped and exhibit a relatively high level of crystallinity (Mishra et al., 2018b). Moreover, cellulose nanocrystals are considered the most resilient and rigid natural nanocellulose available, with valuable properties including high hardness, high strength and high surface area (Lam et al., 2012). However, the disadvantage of nanocrystalline cellulose is its thermal properties in terms of its thermoplasticity due to degradation at around 200–300°C (Brinchi et al., 2013).

It is also worth mentioning that toxicity level studies, also including *in vitro* tests, for both bacterial cellulose, nanofibers as well as cellulose nanocrystals, found no side effects when culturing human endothelial cells, fibroblasts, as well as chondrocytes, and lower levels of toxins (Jeong et al., 2010; Mishra et al., 2018b).

Summarizing the important differences that define possible applications, it is worth emphasizing once again the properties of bacterial cellulose that distinguish it from plant cellulose. First of all, it has higher purity and the absence of hemicellulose, pectin and lignin, greater flexibility and better hydrophilicity, retaining water much better compared to plant-derived nanocellulose (Pecoraro et al., 2008; Amorim et al., 2020). Hence, it has greater economic value. As a nanobiomaterial that is highly chemically reactive and has magnetic and electrical properties that set it apart from other plastics, in the medical industry bacterial cellulose is regarded as one of the plastics of the future. This makes it likely that the use of bacterial cellulose, such as in medicine, will grow every year. Its advantage is the economical way of obtaining it, which does not require the use of specialized equipment. The medical industry appreciates that it is hypoallergenic and biofunctional. It also responds well to the body, which treats it as a natural component of the entire system after implantation.

However, as this review shows, both bacterial and plant cellulose can be used for many applications due to their properties, versatility and sustainable production. As mentioned earlier, research on bacterial cellulose is growing by the day due to its singular properties and greater simplicity in production than that of plant nanocellulose. Certainly, the choice of which cellulosic raw material is more suitable for a given purpose, with respect to the advantages and limitations of a particular type of nanocellulose, will depend on the desired characteristics of the end product and the production requirements.

For a more comprehensive point of view, Table 2 compares the characteristics discussed of bacterial and plant cellulose.

## Conclusion

In recent decades, many studies have been conducted on the modification and use of cellulosic materials. This review focuses on two types of cellulose from different sources, namely from bacterial and plant origin.

A brief characterization and review of these materials with emphasis on their specific properties, such as structure, biochemical and biophysical properties, is made, with further emphasis on their versatility for applications in the biomedical field as well as in the field of polymer composite technology and processing, with particular emphasis on the applicability of biomaterials in the packaging industry, considered the largest consumer sector today.

Although significant progress has been made in the field of tissue engineering, it appears that there are still no materials that fully reproduce the intricacies of native tissue or restore its function to an ideal level. In order to recreate fully functional tissue, the biochemical and biophysical properties must be designed from the nanoscale up. Therefore, it seems that the remaining challenges will be to develop new composite materials using nanoscopic engineering methods to create fully biomimetic tissues.

In order to achieve a compromise between sustainability principles and polymer technology, it is very important to use environmentally friendly materials, therefore biodegradable and compostable packaging made of bioplastics is today an example of an ecological alternative to plastic films and fits perfectly into the principles of the Closed-Loop Economy. Evidently, the fundamental benefits of biocomposites as alternative materials to “petroleum-based” products are certainly shaping current development trends and guiding future research and applications of polymer composites with plant-based raw materials also in many different economic sectors, especially in the automotive and construction industries. Nonetheless, there is still a significant potential to improve the produced composites as well as to improve the efficiency of their manufacturing. It is anticipated that in a future, researchers are likely to focus on nanofillers in biocomposite materials, and to explore more industrialized and efficient processes that are generally difficult to perform with nanotechnology.

For all these reasons, we believe that still some areas that need to be handled and many opportunities that need to be explored remain in this topic.
